# PERSPECTIVE: Treatment with hydrocortisone modified-release capsules in children and adolescents with congenital adrenal hyperplasia: an expert opinion

**DOI:** 10.1530/EC-24-0619

**Published:** 2025-03-26

**Authors:** U Neumann, O Blankenstein, H L Claahsen-van der Grinten

**Affiliations:** ^1^ Charité Universitaetsmedizin Berlin, Clinic for Pediatric Endocrinology and Diabetology, Berlin, Germany; ^2^ Amalia Childrens Hospital, Radboud University Medical Centre, Department of Pediatric Endocrinology, Nijmegen, The Netherlands

**Keywords:** CAH, glucocorticoid treatment in childhood, hydrocortisone modified-release capsules

## Abstract

Recommended treatment for classic congenital adrenal hyperplasia (CAH) in children is hydrocortisone. This therapy is intended to replace cortisol deficiency, but also to reduce the increased production of androgens and their precursors. The aim is to minimize the undesirable side effects of both cortisol deficiency and androgen excess. Short-acting conventional immediate-release hydrocortisone formulations does not mimic physiological diurnal rhythm and often complicate therapy adjustment, mandating frequent administration of often supraphysiological doses, typically 3–4 times daily, including nocturnal dosing. To simulate the physiological diurnal cortisol pattern, a delayed- and sustained-release hydrocortisone preparation has been developed and its efficacy was validated through phase 2 and 3 trials in adult patients. Regulatory approval has been extended to encompass both adult and adolescent patients aged 12 years and older. This manuscript aims to provide treatment principles formulated by two expert centers specialized in pediatric CAH therapy regarding the utilization of recently registered hydrocortisone modified-release capsules in the daily management and stress dosing regimen for adolescents with CAH. It elucidates proposed dosing strategies, therapeutic surveillance protocols and the prospective accumulation of data for the assessment of treatment efficacy during childhood.

## Background and basic principles in diagnostics and treatment

Congenital adrenal hyperplasia (CAH) includes a group of rare inherited adrenal diseases caused by a deficiency of one of the enzymes involved in the adrenal steroid synthesis leading to cortisol deficiency and (in most cases) aldosterone deficiency (1). The compensatory elevation in adrenocorticotropic hormone (ACTH) secretion by the pituitary gland results in adrenal cortex stimulation and subsequent accumulation of steroid hormone precursors preceding the enzymatic deficiencies. The 21-hydroxylase deficiency (21OHD) is the most common enzymatic defect, constituting about 90% of CAH cases (1). The present work focuses on 21OHD, characterized by elevated concentrations of adrenal androgens, such as androstenedione, and specific elevated precursors, such as 17-hydroxyprogesterone (17OHP) and 21-deoxycortisol (21DF). In clinical contexts, 21OHD is typically categorized into three forms based on the residual enzymatic defect, although the clinical spectrum is more fluent. First, the most severe form, classic CAH or salt-wasting CAH (SW-CAH), is characterized by complete absence of enzymatic activity. Second, the classic simple virilizing form (SV-CAH) exhibits residual enzymatic activity ranging from 1 to 2% that is mostly enough for sufficient aldosterone production. Finally, the non-classic CAH (NC-CAH) form manifests with a less severe phenotype due to a residual enzymatic activity of 30–50%, often presenting with mild or asymptomatic features of androgen excess (2). Diagnosis of 21OHD involves assessing elevated adrenal precursors such as 17OHP and 21DF and adrenal androgens such as androstenedione. Many countries include CAH screening in their neonatal screening programs through measurement of 17OHP on filter paper (3).

In contrast to other forms of adrenal insufficiency, treatment of 21OHD consists of replacement therapy of glucocorticoid and potentially mineralocorticoid administration, and second, the reduction of ACTH-mediated androgen excess. The goal of treatment is to get adequate suppression of the adrenal cortex (1). For children with 21OHD, therapy with hydrocortisone given thrice to four times daily is recommended due to its shorter half-life and its reduced impact on growth compared to high-potency glucocorticoids (1). Immediate-release hydrocortisone is generally given as tablets, capsules or suspension. Treatment in NC-CAH depends on symptoms due to the increased adrenal stimulation and overproduction of androgens. Although there is generally no clinically relevant glucocorticoid deficiency in daily life, treatment with glucocorticoids might be necessary during periods of illness. Furthermore, during childhood, elevated androgens may cause an increase in height velocity, with accelerated bone age, and finally reduced final height (4). In women with NC-CAH who desire pregnancy, treatment with glucocorticoids can regularize menstrual cycle and normalize progesterone levels (4).

## Limitations in conventional glucocorticoid treatment in children/adolescents

The recommended treatment regimen for children and adolescents is to administer hydrocortisone at a dosage of 10–15 mg/sqm body surface area (BSA)/day. This dosage exceeds the physiological cortisol production rates in healthy individuals, which is reported to be about 5–8 mg/sqm BSA/day (5) as supraphysiological dosages are mostly necessary to suppress adrenal androgen production.

Physiologically, cortisol production follows a circadian rhythm, peaking in the early morning hours, declining throughout the day and reaching minimal levels in the late evening (6). In 21OHD, achieving physiologic cortisol levels and effectively suppressing the hypothalamic–pituitary–adrenal axis and adrenal androgen production proves challenging with current immediate-release hydrocortisone formulations. These formulations induce fluctuations in glucocorticoid levels, necessitating frequent dosing or employing alternative dosing regimens, such as a nighttime peak dosing schedule (7, 8).

It is imperative to avoid hydrocortisone dosages exceeding 17 mg/sqm/day due to their adverse impact on growth (9) and potential long-term complications such as cardiovascular diseases (10, 11, 12). Despite advancements in care, achieving optimal growth remains a challenge for many children with 21OHD. Notably, there exists a considerable variability in hydrocortisone sensitivity depending on individual characteristics and age periods (13), necessitating careful monitoring of dosing regimens. The procedure for therapy monitoring differs from center to center. One way is to take frequent measurements of salivary 17OHP and androstenedione levels at different times throughout the day to tailor treatment to individual needs (1, 8).

## New treatment modalities in CAH

In recent years, novel preparations and therapeutic approaches have emerged with the principal objective to mimic physiologic cortisol levels (for figure see (6)) and to reduce adrenal androgen levels in patients with 21OHD (14). Modified-release hydrocortisone formulations are designed to delay hydrocortisone release, thereby suppressing ACTH levels more efficiently, particularly during the early morning hours (15). Two such formulations have been developed: Plenadren^®^ (Shire Pharmaceuticals Ireland Limited, Ireland), featuring a dual-release mechanism, and Efmody^®^ (Chronocort) – hydrocortisone modified-release capsules (hydrocortisone MRC) (Diurnal, UK), characterized by delayed- and sustained-release (16).

Plenadren^®^ is not considered suitable for CAH patients due to its once-daily morning administration, which fails to adequately suppress the early morning surge of ACTH and adrenal androgens in 21OHD (17, 18). Conversely, hydrocortisone MRC has been specifically tailored to address the nocturnal rise of ACTH and thereby the elevated androgen levels during this period in CAH (16).

## Background of hydrocortisone modified-release capsules (Efmody^®^/Chronocort)

Since its authorization by the EMA in May 2021, hydrocortisone MRC, a controlled-release cortisol preparation, has become available in various European countries, including Germany, since September 2021, and the Dutch market since March 2022. It is approved for adults and adolescents aged 12 years and older and is offered in two dose strengths: 5 and 10 mg in hard capsules. Utilizing a multiparticulate formulation involving drug and polymer-layering techniques, hydrocortisone MRC uses a delayed-release technology: an inert core coated with hydrocortisone is enveloped in a pH-sensitive layer, facilitating delayed drug release in the distal small intestine at a pH of 6.8 (19).

Compared to conventional immediate-release hydrocortisone medication, hydrocortisone MRC exhibits a delayed time to maximum cortisol concentration (Tmax), ranging from 4.5 to 6.75 h post-ingestion (in fasting and fed states, respectively), in contrast to the typical Tmax of 1 h for immediate-release hydrocortisone. This delay in Tmax allows for better alignment with the physiological diurnal rhythm of cortisol secretion, with the highest concentration observed in the early morning hours, commencing at 1–2 h, although medication is taken at bedtime. This aims to mitigate the overnight rise of ACTH and the synthesis of adrenal androgens during this time period (19).

Treatment with hydrocortisone MRC at a twice-daily dose has demonstrated normalization of 17OHP and androstenedione levels and reduction in urinary 17OHP and androgen metabolite excretion in adults compared to therapy with conventional immediate-release hydrocortisone, despite a reduction in total daily glucocorticoid dose (15, 20, 21). Quality of life (QoL) and fatigue scores at baseline were similar to those of a healthy population and did not change after 6 months hydrocortisone MRC treatment. Notably, one male patient with a history of testicular adrenal rest tumors (TARTs) experienced an improved sperm count during therapy with hydrocortisone MRC (21).

Pharmacokinetic modeling suggests that physiological cortisol levels in adults are best achieved with a dose combination of two-thirds total dose last thing at night and one-third in the morning, for example, 15 or 20 mg at 23:00 h and a smaller dose of 10 mg at 07:00 h (15). Pharmacokinetic studies provides similar suggested dosing for adolescents aged 12–18 years (22), although this dosage has not yet been investigated in childhood studies and the calculation in childhood should be made in relation to BSA.

During puberty, increased clearance of cortisol is observed due to decreased activity of 11β-hydroxysteroid dehydrogenase type 1 (11β-HSD1), pubertal insulin resistance and increased clearance by the kidney, necessitating higher glucocorticoid doses during this period (23). Doses exceeding 17 mg/sqm BSA/day should be avoided due to their negative impact on final height (9). There is a lack of clinical data and phase 2 and 3 studies on the use of this medication in adolescents below 18 years of age.

Research questions include whether adequate hormonal control can be achieved or improved with similar daily dosages as conventional hydrocortisone treatment with a twice-daily dosing regimen of hydrocortisone MRCs, and the effect on final height, fludrocortisone dosages, therapy adherence and QoL and any potential side effects. Long-term outcomes such as the development of TARTs in males, menarche in females and cardiovascular risk profiles also warrant investigation. A standardized treatment protocol is suggested to address these research questions.

## Recommendations for treatment with hydrocortisone MRC in children and adolescents aged 12 years and older

In the absence of dedicated studies for hydrocortisone MRC in this age, dosing recommendations align with established guidelines for childhood adrenal insufficiency/CAH. These recommendations adapt the dosing regimen from adult therapy, dividing the total daily dose in two administrations: approximately two-thirds to three-quarters in the evening (at bedtime, at least 2 hours after the evening meal) and the remainder in the morning upon awakening (1 hour before breakfast). This approach is supported by pharmacokinetic modeling in a virtual pediatric population aged 12–18 years, which showed comparable results to adult data and predictable pharmacokinetics in a pediatric population (22). Hydrocortisone MRC treatment in adolescents or even younger patients is limited by available dose strengths of hydrocortisone MRC in 5 and 10 mg capsules. Single doses and dose titration are only possible in steps of 5 mg, while during childhood, capsules of 2.5 mg dose would be very helpful for accurate dose adjustment with the lowest possible dose.

## Proposed treatment protocol

### Start treatment with hydrocortisone MRC

Hydrocortisone modified-release capsules are licensed for treatment of CAH in children older than 12 years and adults. Actually, only 5 and 10 mg capsules are available, which cannot be split. Conversion can therefore only be completed in increments of 5 mg. We advise to convert conventional treatment with hydrocortisone according to the table below (Table 1).

**Table 1:** Dose adjustment of hydrocortisone after change to hydrocortisone MRC.

Daily dose hydrocortisone (mg)	Daily dose hydrocortisone MRC (mg)	Morning dose hydrocortisone MRC upon awakening (mg) (at least 1 h before breakfast)	Evening dose hydrocortisone MRC at bedtime (mg) (at least 2 h after last meal)
28–32	30	10	20
23–27.5	25	10	15
18–22.5	20	5	15
	or	10	10
15–17.5	15	5	10

### Adjusting hydrocortisone MRC during treatment

Regular therapy monitoring is essential for adjusting hydrocortisone MRC dosage. According to the current guidelines, individualized monitoring with timed blood or saliva sampling is recommended, preferably with multiple timepoints throughout the day taken just before the next hydrocortisone dose (1, 3). If adrenal androgen levels exceed the reference range, the preceding hydrocortisone MRC dose should be increased by 5 mg increments every 3 months. Conversely, if adrenal androgens are suppressed, preceding hydrocortisone MRC dose should be decreased every 3–6 months in 5 mg increments. During the day, it is also practical to take a dose of immediate-release hydrocortisone, e.g., at lunchtime or in the early afternoon, if the dose of hydrocortisone MRC in the morning is not sufficient. An additional dose of immediate-release hydrocortisone during the night or early morning hours would cancel out the effect of an undisturbed sleep under hydrocortisone MRC.

### Follow-up during treatment

The parameters summarized in Table 2 should be monitored in patients treated with hydrocortisone MRC (Table 2).

**Table 2:** Recommended examination of patients with CAH after switching therapy to hydrocortisone modified-release capsules; measurement in males (m) or females (f).

Parameters	T0	3-monthly	6-monthly	Yearly
Height	X	X		
Weight	X	X		
RR	X	X		
Tanner stage	X		X	
Testicular volume (mL)	X		X	
17OHP*	X	X		
Androstenedione*	X	X		
Renin	X			X
LH	X			X
FSH	X			X
Testosterone	X			X
Estradiol (f)	X			X
Inhibin B (m)	X			X
Sodium	X			X
Potassium	X			X
Ultrasound testes (m)	X			X
Bone age until final height	X			X
History of stress dosing	X	X		
History of adrenal crisis	X	X		
History of menstruation (f)	X	X		

* Saliva or serum samples are collected in the morning and in the evening immediately before hydrocortisone medication is taken.

Abbreviations: RR, blood pressure measurement by method of Riva-Rocci; 17OHP, 17-hydroxyprogesterone.

### Stress dosing during treatment with hydrocortisone MRC

Due to the delayed- and sustained-release preparation, hydrocortisone MRC are not suitable for acute stress treatment and immediate-release hydrocortisone should be used following standard schemes within the countries (3–4 fold of daily hydrocortisone dosing given in 3–4 doses/24 h). Besides, we recommended continuing treatment with hydrocortisone MRC treatment also during (short-term) stress dosing, as specified in the medication leaflet. In serious situations, the dose should be increased immediately and the oral application of hydrocortisone should be replaced by parenteral treatment. Intramuscular hydrocortisone preparations are available for this purpose.

### Data collection

Given the absence of clinical trials in children and adolescent, prospective data collection is essential to evaluate a broader cohort over time, facilitating improved recommendations in the future. For comprehensive data collection, we recommend the utilization of international SDM registries (https://sdmregistries.org/). Long-term data on growth, body weight, pubertal development and biochemical control give the opportunity to investigate the treatment outcome in different ages during childhood and also in different forms of CAH, e.g., non-classic CAH versus classic CAH, and in different centers of expertise from all over the world. The assessment of health-related QoL is not yet implemented. We recommend that such a validated questionnaire for childhood and adolescence, and questions on general QoL, be included in the registry.

## Conclusion

Hydrocortisone MRC has been authorized by the EMA since 2021 for the treatment of CAH in children older than 12 years and adults. The delayed-release of hydrocortisone MRC can mimic the physiological rise in cortisol in the early morning hours and consequently lowers levels of ACTH, androgens and steroid hormone precursors in the morning.

Controlled trials assessing treatment outcomes in children and adolescents are currently lacking. Here, we provide recommendations for the use of hydrocortisone MRC based on clinical experience and results from adult studies. Prospective, standardized data collection is warranted to investigate long-term effects on growth, weight gain, pubertal development, onset of menarche in females, development of TART in males, metabolic parameters, bone health and general well-being.

## Declaration of interest

OB and UN’s employee received clinical trial fee by Neurocrine Inc. UN received a lecture fee and travel grant of Diurnal Ltd, a lecture fee of Novo Nordisk Pharma GmbH and a lecture fee and travel grant of Merck-Healthcare Germany GmbH. OB received a travel grant from Novo Nordisk Pharma. HC received clinical trial fee from Spruce Biosciences.

## Funding

This work did not receive any specific grant from any funding agency in the public, commercial or not-for-profit sector.
